# Cystic lymphangioma of the adrenal gland: report of a case and review of the literature

**DOI:** 10.1186/s12957-015-0490-0

**Published:** 2015-02-15

**Authors:** Gaëtan-Romain Joliat, Emmanuel Melloul, Reza Djafarrian, Sabine Schmidt, Sara Fontanella, Pu Yan, Nicolas Demartines, Nermin Halkic

**Affiliations:** Division of Visceral Surgery, University Hospital CHUV, Rue du Bugnon 46, 1011 Lausanne, Vaud, CH Switzerland; Department of Radiology, University Hospital CHUV, Lausanne, Switzerland; Department of Pathology, University Hospital CHUV, Lausanne, Switzerland

**Keywords:** Cystic lymphangioma, Retroperitoneal tumor, Adrenal tumor, Cystic lesion

## Abstract

**Background:**

Cystic lymphangioma is a rare tumor of the lymphatic vessels that occurs more frequently in women. Location of this pathology can be diverse but most commonly occurs in the neck or axilla. Cystic lymphangioma originating from the adrenal tissue represents a very rare entity.

**Case presentation:**

We report here the case of a 38-year-old woman who was diagnosed with a cystic retroperitoneal mass. After further investigations, the patient was suspected to have a left adrenal cystic lymphangioma. She underwent successful open left adrenalectomy as curative treatment, and the diagnosis of cystic lymphangioma of the left adrenal gland was confirmed at histology. The postoperative course was uneventful.

**Conclusion:**

This case report and review of the literature bring new insights into the diagnostic difficulty and management of cystic lymphangioma of the adrenal gland.

## Background

Cystic lymphangiomas are rare benign lesions of the lymphatic vessels [1]. These tumors originate from the lymphatic endothelial cells and are thought to be due to ectasia or abnormal development of lymphatic vessels [2-4]. Most of the time, these tumors appear in the neck or axilla [1], whereas intra-abdominal lymphangiomas only account for 5% of all lesions [2]. Adrenal cysts in general are uncommon entities occurring in about 0.06% of the population [5], in which cystic lymphangiomas account for a minority of cases. The majority of cystic lymphangioma cases were described in women [2,6-13]. Therefore, the rarity of this disease and the lack of report render the diagnosis and management of this entity challenging. We report a case of left adrenal cystic lymphangioma in a female patient and review the current literature.

## Case presentation

A 38-year-old woman known for anxiety disorder developed constant epigastric pain without radiation toward the back. She mentioned that the pain was not related to the food intake and scored it between four and six (out of ten) on a visual analog scale. She had multiple episodes of vomiting, no bowel movement problems, and no fever. Her past medical and surgical history was otherwise uneventful. She went to her general practitioner who first performed an ultrasonography (US) and a CT scan. These exams showed a cystic-like retroperitoneal mass on the left side measuring 13.4 × 7.2 × 5.2 cm. Laboratory tests were normal. No further exams were undertaken at this point. The pain slightly diminished with paracetamol and non-steroidal anti-inflammatory drugs. However, after a couple of months, the pain reappeared, and the patient was scheduled for a CT-guided puncture of this cyst.

**Figure 1:**
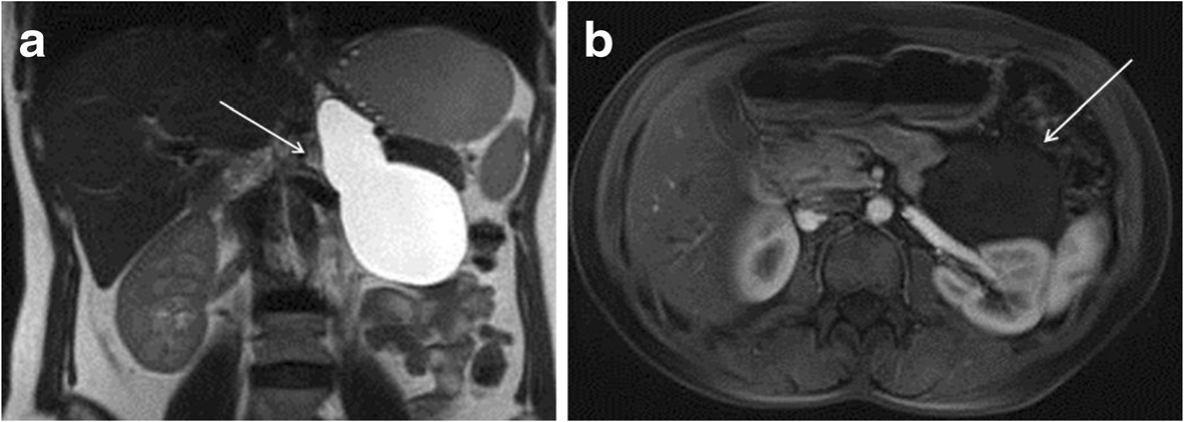
**Magnetic resonance (MR) imaging of the cystic lymphangioma.** Coronal **(a)** T2-weighted turbo spin-echo and axial **(b)** T1-weighted contrast-enhanced gradient-echo MR images show an ovoid left adrenal lesion (arrow). It is homogeneously hyperintense on T2-weightening **(a)** and hypointense on T1-weightening **(b)**, thus confirming the fluid content without loculation neither solid component. The thin wall surrounding the lesion is barely perceptible.

At first, the radiologist was not able to puncture the cyst due to a thick capsule. The cyst was finally punctured under US control but could not be completely evacuated due to technical problems (dysfunctional guide wire). Cytology came back negative for malignant cells and was compatible with a cystic lymphangioma. Unfortunately, the puncture of the cyst did not help to relieve the patient’s symptoms. The patient then underwent a magnetic resonance imaging (MRI) to assess more precisely the location of this retroperitoneal cyst and its anatomic relations. T2-weighted turbo spin-echo MR-sequences showed a lesion compatible with a cystic lymphangioma originating from the left adrenal gland (Figure 1). The cystic lesion extended inferiorly to the renal vein and superiorly to the diaphragmatic pillar. Due to persisting invalidating pain, a surgical resection of this cystic lesion was proposed. The patient accepted the operation and signed the informed consent form.

Due to the size and position of the cyst, and to avoid a rupture of the cyst during laparoscopy, a left subcostal laparotomy was performed. During surgery, the adrenal origin of the cyst was confirmed, and an ‘*en bloc*’ left adrenalectomy was performed without rupture of the cyst. No drain was left in place. The postoperative course was uneventful, and the patient was discharged on postoperative day 5. The patient was seen at the outpatient clinic one month after surgery and described no more symptoms.

**Figure 2:**
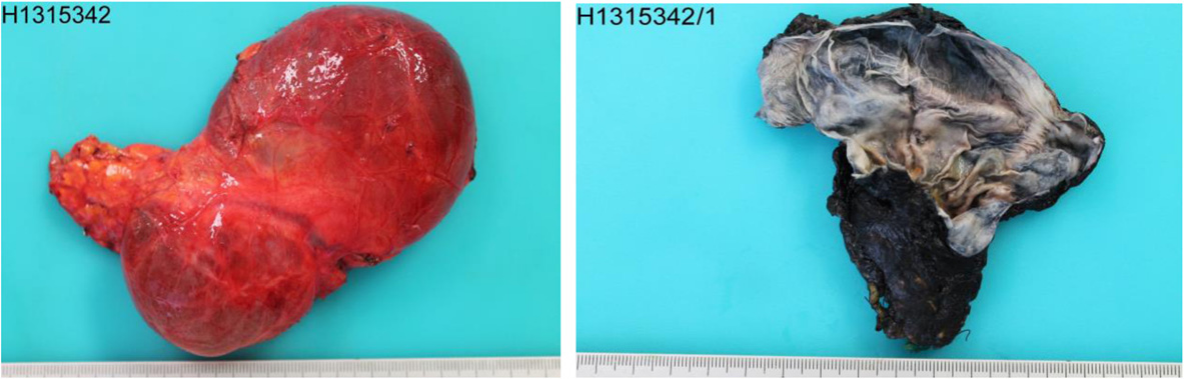
**Pathological macroscopic image.** Macroscopic views of the resected adrenal specimen measuring 8.5 × 4.3 × 2.8 cm.

**Figure 3:**
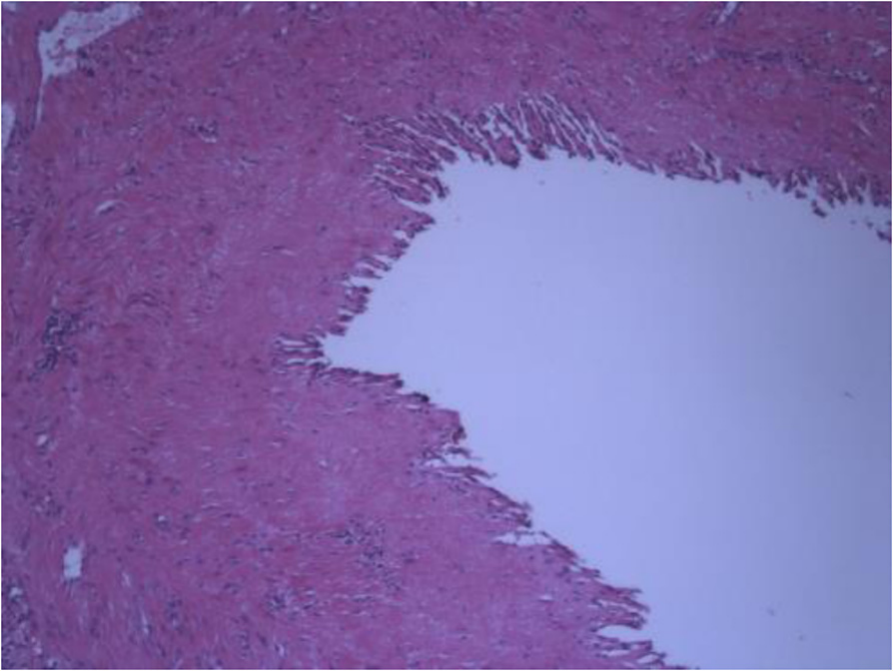
**Histological view of the cystic lymphangioma of the left adrenal gland (hematoxylin and eosin staining, 40×).**

**Figure 4:**
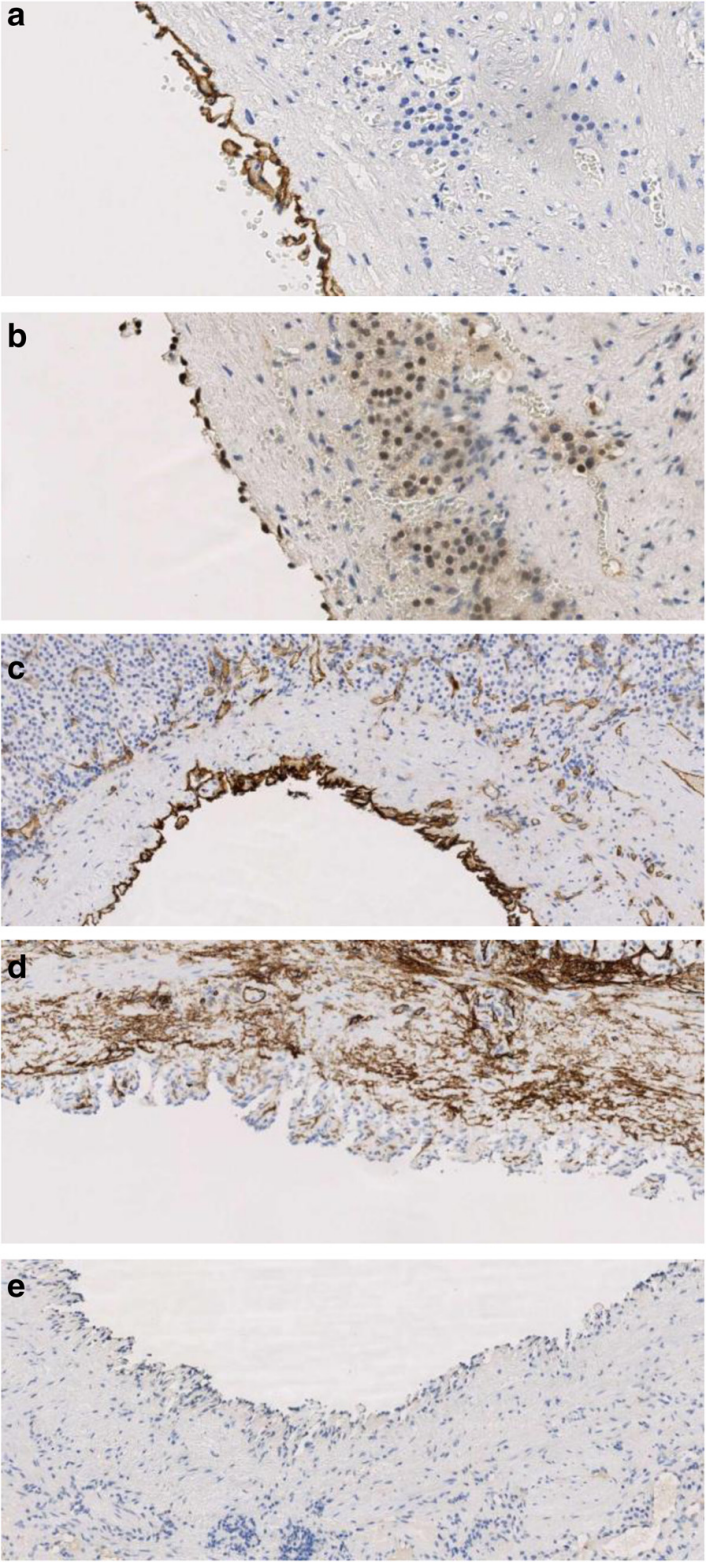
**Immunohistochemistry images.** D2-40 **(a)**, PROX1 **(b)**, CD31 **(c)**, CD34 **(d)**, and CKAE1/AE3 **(e)** immunohistochemical stains.

The specimen consisted of an ovoid, cystic mass measuring 8.5 × 4.3 × 2.8 cm (Figure 2). An unremarkable adrenal gland, measuring 3 × 1.2 × 0.7 cm, partially surrounded the cyst. The inner and outer surfaces of the cyst were smooth with no evidence of rupture. The wall of the lesion was thin with no tumor excrescences. The cyst was filled with clear fluid. Hematoxylin and eosin stain showed a cystic space lined by a single layer of flattened cells, with occasional pseudopapillae formation and bands of smooth muscle in the wall (Figure 3). The lining cells had oval, regular nuclei and showed no atypia. The lining cells showed strong immunoreactivity for D2-40, PROX1, and CD31 and absence of staining for CD34 and CKAE1/AE3 (Figure 4). The diagnosis of cystic lymphangioma originating from the left adrenal gland was then confirmed.

### Discussion

This article reports a rare case of symptomatic cystic lymphangioma originating from the left adrenal gland in a female patient successfully treated by complete surgical resection.

The majority of intra-abdominal cystic lymphangiomas is located in the mesentery, in contrast to adrenal location that is very rare [12]. No more than 30 cases of adrenal cystic lymphangioma have been described in the literature [2,3,6-14]. Table 1 summarizes the English-written cases reported in the literature since 2000. As confirmed in this case report, age at symptom onset usually ranges from 30 to 50 years with a peak incidence during the fourth decade [4,10-12,14]. Cystic lymphangioma can occur in both adrenals [14], but the right side is more often affected [2]. It also occurs more frequently in women [2].

**Table 1:** **Cases of cystic lymphangioma of the adrenal gland reported in the English-written literature from 2000**

	**Authors**	**Publication year**	**Patient number**	**Gender**	**Age**	**Open (O) or laparoscopic ablation (L)**	**Symptom related to lymphangioma**	**Left/right adrenal gland**
1	Akand et al. [6]	2013	1	F	44	O	Pain	L
2	Sourial et al. [7]	2013	1	F	52	L	*Nihil*	L
3	Makni et al. [12]	2012	2	F/M	40/40	O/L	*Nihil*/pain	2 L
4	Ellis et al. [2]	2011	9	6 F/3 M	28 to 56^a^	Not precised	5 *Nihil*/4 pain	6 R/3 L
5	Chien et al. [13]	2008	8	6 F/2 M	31 to 59^a^	Not precised	4 *Nihil*/2 pain/1 fever/1 HTN^b^	4 R/4 L
6	Bettaïeb et al. [8]	2007	1	F	22	Not precised	Pain	L
7	Ates et al. [9]	2005	1	F	26	O	Weakness	R
8	Garcia et al. [10]	2004	1	F	22	Not precised	Pain	R
9	Longo et al. [11]	2000	1	F	30	O	Pain	R
10	Trojan et al. [3]	2000	1	M	40	Not precised	*Nihil*	R

^a^Range; ^b^hypertension.

Of note, lymphangioma is the generic term for a tumor arising from the lymphatic vessels and is often found in children. Lymphangiomas have an endothelial origin. The exact pathogenesis is currently not completely elucidated, and whether lymphangioma of the adrenals is a real neoplasm remains unclear [2]. The most likely etiology is a developmental abnormality or ectasia of the lymphatic vessels [3,4]. Cystic lymphangioma develops when a blockage of the lymphatic vessels occurs due to a benign proliferation. It should not be confused with lymphangioma-like adenomatoid tumors which have a different embryonic origin [2].

Differential diagnosis of a retroperitoneal cystic-like lesion includes primary adrenal tumors, metastatic adenocarcinomas, angiosarcomas, multicystic mesotheliomas, or adrenal cysts [9]. Adrenal cysts can be further subdivided into pseudocysts, endothelial cysts (lymphangiomatous or angiomatous), and epithelial cysts [4]. Most of the time, lymphangiomas are non-secreting and are discovered incidentally during a radiological exam or a surgery. Symptomatic tumors can induce pain, fever, gastrointestinal disturbances, or hypertension [4,13]. Complications of this kind of tumors mainly are enlargement-causing pain or hemorrhage into the cyst. Diagnostic suspicion is based on clinical presentation, radiological images, and cytological exams.

Cystic lymphangioma of the adrenals does not have a pathognomonic radiological presentation, but new imaging modalities bring useful information helping the diagnosis [15]. As the lesion is rare in this organ, the radiological images lack specificity. On US, adrenal lymphangioma appears as an anechoic lesion in the suprarenal location [2,11]. US can be a good first exam modality [12]. Usually adrenal lymphangioma appears hypodense with smooth borders on CT scanner [16]. On MRI, cyst borders are delineated by injection of contrast. T1- and T2-weighted MR images are not pathognomonic, but adrenal lymphangioma usually appears as hypointense on T1-weighted sequences and homogeneously hyperintense on T2-weighted sequences. MRI being far more specific than CT, it usually allows distinguishing malignant adrenal lesions from benign ones [15]. Differential diagnosis includes metastatic tumors, carcinomas, or pheochromocytomas. Moreover, MRI is more sensitive than CT scan to detect degeneration of the cyst or intracystic hemorrhage [6,16].

Immunohistochemistry is an important tool to differentiate this pathological entity from other diagnoses. Lymphangiomas usually display D2-40, PROX1, and CD31 positivity and absence of CD34 and CKAE1/AE3 stains [2]. Final diagnosis is made by histopathology combined with immunohistochemistry [12].

Asymptomatic cystic lymphangioma discovered incidentally can just be followed clinically or with control imaging, as there is no risk of malignant degeneration [12]. Puncture of the cyst can help the diagnosis but is not a therapeutic measure, as a punctured cystic lymphangioma will recur rapidly as demonstrated in this case. Puncture with injection of sclerosing agents like bleomycin have been tried but showed the same recurrence risk [12]. Surgical resection represents the definitive treatment if the cystic lymphangioma is symptomatic. Complete resection of the cyst is recommended, and associated parenchymal resection (adrenalectomy) depends on the location of the cyst and on the intraoperative dissection [12]. Decision to undertake a laparotomy or a laparoscopy depends on the position, the size, and the risk of rupture of the cyst. No data on the recurrence risk if intraoperative cyst perforation occurs are currently available in the literature. If the cystic lymphangioma is bleeding, preoperative embolization can also be considered [12].

## Conclusions

In summary, cystic lymphangioma of the adrenal gland is a rare pathology that should be included in the differential diagnosis of cystic lesions of the adrenal glands. Its diagnosis can be difficult and challenging. MRI seems to be a good diagnostic modality to detect degeneration or intracystic hemorrhage. If the patient is symptomatic, definitive treatment is surgery.

## Consent

Written informed consent was obtained from the patient for publication of this case report and any accompanying images. A copy of the written consent is available for review by the Editor-in-Chief of this journal.

## Abbreviations

CT: computed tomography
MRI: magnetic resonance imaging
US: ultrasonography
